# Intrinsic Features in MicroRNA Transcriptomes Link Porcine Visceral Rather than Subcutaneous Adipose Tissues to Metabolic Risk

**DOI:** 10.1371/journal.pone.0080041

**Published:** 2013-11-06

**Authors:** Jideng Ma, Zhi Jiang, Shen He, Yingkai Liu, Lei Chen, Keren Long, Long Jin, An'an Jiang, Li Zhu, Jinyong Wang, Mingzhou Li, Xuewei Li

**Affiliations:** 1 Institute of Animal Genetics and Breeding, College of Animal Science and Technology, Sichuan Agricultural University, Ya'an, Sichuan, China; 2 Novogene Bioinformatics Institute, Beijing, China; 3 Chongqing Academy of Animal Science, Chongqing, China; ENEA, Italy

## Abstract

MicroRNAs (miRNAs) are non-coding small RNA ∼22 nucleotides in length that can regulate the expression of a wide range of coding genes at the post-transcriptional level. Visceral adipose tissues (VATs) and subcutaneous adipose tissues (SATs), the two main fat compartments in mammals, are anatomically, physiologically, metabolically, and clinically distinct. Various studies of adipose tissues have focused mainly on DNA methylation, and mRNA and protein expression, nonetheless little research sheds directly light on the miRNA transcriptome differences between these two distinct adipose tissue types. Here, we present a comprehensive investigation of miRNA transcriptomes across six variant porcine adipose tissues by small RNA-sequencing. We identified 219 known porcine miRNAs, 97 novel miRNA*s, and 124 miRNAs that are conserved to other mammals. A set of universally abundant miRNAs (i.e., miR-148a-3p, miR-143-3p, miR-27b-3p, miR-let-7a-1-5p, and miR-let-7f-5p) across the distinct adipose tissues was found. This set of miRNAs may play important housekeeping roles that are involved in adipogenesis. Clustering analysis indicated significant variations in miRNA expression between the VATs and SATs, and highlighted the role of the greater omentum in responding to potential metabolic risk because of the observed enrichment in this tissue of the immune- and inflammation-related miRNAs, such as the members of miR-17-92 cluster and miR-181 family. Differential expression of the miRNAs between the VATs and SATs, and miRNA target prediction analysis revealed that the VATs-specific enriched miRNAs were associated mainly with immune and inflammation responses. In summary, the differences of miRNA expression between the VATs and SATs revealed some of their intrinsic differences and indicated that the VATs might be closely associated with increased risk of metabolic disorders.

## Introduction

MicroRNAs (miRNAs) are a family of small single-stranded non-coding RNAs, which are known to function in a sequence-specific manner to silence specific protein-coding genes at the post-transcriptional level by targeting the 3′ untranslated region of mRNAs [1]. With the rapid increase in knowledge that has accumulated over the last decade, various miRNAs have been shown to play vital regulatory roles in adipose deposition and adipogenesis. Typically, miR-143 is a potent pro-adipogenic regulator during pre-adipocyte differentiation [2], [3], [4], and the miR-17-92 cluster [5] and miR-103 [6] can accelerate adipocyte differentiation, while miR-27a [7], miR-27b [8] and miR-15a [9] can suppress adipogenic differentiation. In addition, miR-519d [10], miR-335 and miR-377 [11] are strongly associated with lipid metabolism disorders.

Adipose tissues (ATs) are currently recognized as an endocrine organ, and the number of adipokines that have been identified in ATs is expanding rapidly. ATs are deeply involved in the development of metabolic disorders, such as cardiovascular disease and type 2 diabetes mellitus, which are connected to obesity [12], [13], [14]. Nonetheless, the different fat compartments may be associated with differential metabolic risk. Visceral adipose tissues (VATs), which are located within the abdominal and thoracic cavities, have been recognized to be more strongly associated with metabolic risk factors than the subcutaneous adipose tissues (SATs) [15], [16], [17]. It was suggested that the different impacts that VATs and SATs have on metabolic risk may be because of diverse gene expression profiles that lead to differences in lipolysis and in the production and release of adipokines and cytokines [17]. However, miRNA-based gene regulatory mechanisms in the distinct ATs are yet to be investigated. The results will be of interest for the development of diagnostics and therapeutics for metabolic diseases.

Pigs have considerable agricultural significance and are an important model system for human biomedical research, including the study of obesity and energy metabolism [18]. To explore the molecular mechanisms that underlie the metabolic and functional differences between SATs and VATs, we performed a comprehensive comparison of the miRNA transcriptomes from six types of porcine ATs and identified various known and conserved miRNAs. We found that the SATs-specific enriched miRNAs were associated primarily with lipid metabolic homeostasis, whereas the VATs-specific enriched miRNAs were related mainly to the immune and inflammatory responses, which indicated the metabolic risk roles of the VATs. We envision that these findings will contribute to the further understanding of the biological functions of miRNAs in ATs, and the molecular mechanisms behind the distinct metabolic and physiological roles of the SATs and VATs.

## Materials and Methods

### Ethics statement

All research involving animals were conducted according to the Regulations for the Administration of Affairs Concerning Experimental Animals (Ministry of Science and Technology, China, revised in June 2004) and approved by the Institutional Animal Care and Use Committee in College of Animal Science and Technology, Sichuan Agricultural University, Sichuan, China under permit No. DKY-B20110807. Animals were allowed free access to food and water under normal conditions, and were humanely sacrificed as necessary, to ameliorate suffering.

### Animals and sample collection

Three 210-days old female Landrace pigs with normal weight (111.67±1.15 kilograms) were used. A starter diet provided 3.40 Mcal·kg^−1^ metabolisable energy (ME), 20.00% crude protein and 1.15% lysine from the thirtieth to sixtieth day after weaning. From the 61^st^ to the 120^th^ day, the diet contained 3.40 Mcal·kg^−1^ ME, 17.90% crude protein and 0.83% lysine. From the 121^st^ to 210^th^ day, the diet contained 3.40 Mcal·kg^−1^ ME, 15.00% crude protein and 1.15% lysine.

Four VATs (i.e. greater omentum (GOM), mesenteric adipose (MAD), pericardial adipose (PAD) and retroperitoneal adipose (RAD)), and two SATs (i.e. upper layer of back fat (ULB) and inner layer of back fat (ILB)) were rapidly separated from each carcass, immediately frozen in liquid nitrogen, and stored at −80°C until RNA extraction.

### Small RNA libraries construction and deep sequencing

The total RNA of 18 tissue samples were extracted with *mir*Vana™ miRNA isolation kit (Ambion, Austin, USA), and further purified with Rneasy column (QIAGEN, Hilden, Germany). The quantity and purity of total RNA were monitored via analysis by NanoDrop ND-1000 spectrophotometer (Nano Drop, DE, USA) at 260/280 nm (ratio>2.0). The integrity of total RNA was also tested via analysis by Bioanalyzer 2100 and RNA 6000 Nano LabChip Kit (Agilent, CA, USA) with RIN number>6.0.

For a certain adipose tissue, equal amounts (5 µg) of small-RNA-enriched total RNA isolated from three pigs were mixed and prepared for Illumina sequencing. In general, the processing by Illumina consisted of the following successive steps: the small RNA ranged from 14 to 40 nt were purified by polyacrylamide gel electrophoresis (PAGE) and ligated specific adapters followed by polyacrylamide gel purification. Then the modified small RNA was reverse transcripted and amplificated by RT-PCR. Finally, the enriched cDNA was sequenced on Genome Analyzer Instrument (GAI, Illumina). The small RNA sequence data have been uploaded to NCBI's Gene Expression Omnibus (GEO) [accession number GSE30334].

### *In silico* analysis of small RNA-sequencing data

The raw reads were processed using Illumina's Genome Analyzer Pipeline software and subsequently handled as described by Li *et al.* [19], [20] with some improvement. After trimming off the sequencing adapters, the resulting reads was successively filtered by read length (only read length ranged from 14 to 27 nt were retained) and sequence component (containing no more than 80% A, C, G or T; containing no more than two N (undetermined bases)). Given the confidence of analysis results, the resulting set of distinct reads was subsequently filtered by copy numbers (the low-abundance reads (copy number<3) was excluded). Then the retained reads were searched against the NCBI [21], Rfam [22] and Repbase database [23] to remove porcine known classes of RNAs (i.e. mRNA, rRNA, tRNA, snRNA, snoRNA and repeats). The sequencing reads survived from above strict filter rules were deemed to ‘high-quality reads’.

The high-quality reads were mapped to the pig genome (∼2.26 Bbp) (Sscrofa9) using NCBI Local BLAST. The mapping process included two steps: (1) map the high-quality reads to the 228 known porcine pre-miRNAs (encoding 257 miRNAs) and then to 6,716 known pre-miRNAs (encoding 7952 miRNAs) from 24 other mammals in miRBase 18.0 [24]; (2) map the mapped high-quality reads to pig genome to obtain their genomic locations and annotations in Ensembl release 59 (Sscrofa9, April 2009).

### MiRNA differential expression analysis

Program IDEG6 [25] was employed for detecting the differentially expressed miRNAs in the pairwise comparison between the VATs and SATs. If a unique miRNA simultaneously obtains a *P*<10^−5^ under each of the three statistical tests (a Audic-Claverie test, a Fisher exact test and a Chi-squared 2×2 test) with the Bonferroni correction, it should been termed as a differentially expressed miRNA.

### Prediction and functional annotation of miRNA target genes

The potential targets of a certain miRNA were predicted using the PicTar [26], TargetScan human 6.2 [27] and, MicroCosm Targets (version 5.0) [28], and the pairwise overlaps of the results from three programs were composed the final predicted targets. The Gene Ontology-biological process (GO-BP) and KEGG pathway terms enriched in the predicted target genes were determined using a DAVID bioinformatics resources [29].

### Q-PCR validation

The expression changes of 18 selected miRNAs were validated by an EvaGreen-based High-Specificity miRNA qRT-PCR Detection Kit (Stratagene, La Jolla, USA) on the CFX96™ Real-Time PCR Detection System (Bio-Rad, CA, USA). The q-PCR validation were carried out on three biological replicates. The primer pairs were available in Table S1. Three endogenous control genes (porcine U6 snRNA, 18S rRNA and Met-tRNA) [20] were used in this assay. The ΔΔCt method was used to determine the expression level differences between surveyed samples. Normalized factors (NF) of three endogenous control genes and relative quantities of objective miRNAs were analyzed using the qBase software [30].

## Results

### Identification of miRNAs in six distinct ATs

Six small RNA libraries, two SATs (i.e. ULB andILB) and four VATs (i.e. GOM, RAD, MAD and PAD), were constructed and sequenced. We obtained an average of 21.10 million (M) (21.10±5.95 M, *n* = 6) raw reads from each library. More than 70% (77.62±7.15%, *n* = 6) of the raw reads in each library passed the quality filter (see Methods) and were defined as ‘high-quality reads’ (Figure S1A), which is consistent with the canonical size range of mammalian miRNAs (Figure S1B). The vast majority of high-quality reads were 21−23 nt in length (92.04±0.47%, *n* = 6); more than half of them were 22 nt in length (66.45±1.44%, *n* = 6), followed by 21 nt (14.42±1.75%, *n* = 6) and 23 nt (11.17±1.44%, *n* = 6). These reads were selected as the reliable miRNA candidates for subsequent analysis.

A total of 440 mature miRNAs corresponding to 271 miRNA precursors (pre-miRNAs) were identified in the six libraries. The identified precursor and mature miRNAs were divided into two groups using the following alignment criteria (Table S2): (1) Known porcine miRNAs: 316 of the miRNAs mapped to 184 known porcine pre-miRNAs. Specifically, 219 miRNAs were documented in miRBase [28], and 97 were newly identified miRNA*s; (2) Conserved miRNAs: 124 reads mapped to 87 mammalian (other than porcine) pre-miRNAs in miRBase. The conserved miRNAs mapped to the pig genome and were labeled with the names of the corresponding miRNAs. Among them were distinct pre-miRNAs that coded identical mature miRNAs, resulting in the 440 mature miRNAs corresponding to 409 unique miRNA sequences (Table S3).

### Universally abundant miRNAs across distinct ATs

**Figure 1:**
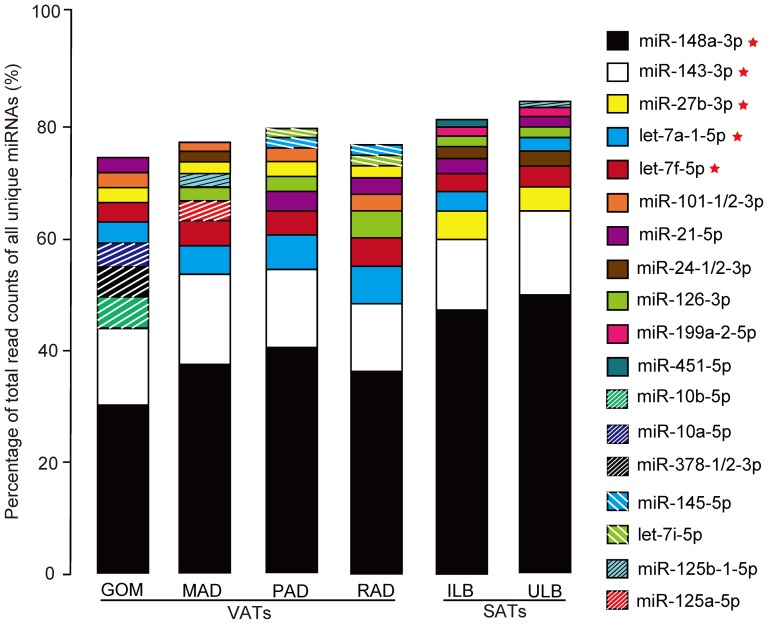
Top 10 unique miRNAs with the highest expression level in six adipose tissues. Five miRNAs that have the highest abundance in all six libraries were marked with the symbol of red stars.

In this study, we observed that the majority of abundant miRNAs were from a few miRNA species. As shown in Figure 1, the top 10 unique miRNAs with the highest expression levels account for more than 74.42%, by total read counts, of the 409 unique miRNAs. The unified set of top 10 unique miRNAs over the six ATs correspond to 18 unique miRNAs, five of which (miR-148a-3p, miR-143-3p, miR-27b-3p, let-7a-1-5p and let-7f-5p) have the highest abundance in all six libraries. These five miRNAs may play potential housekeeping roles in the adipocytes, and hence may be important regulators involved in adipogenesis. For example, miR-148a-3p [31] was reported to be up-regulated during the differentiation of 3T3-L1 pre-adipocyte, whereas miR-27b-3p was suggested to be a negative regulator during adipogenesis [8]. MiR-143 was elevated in adipogenesis and the loss of function of miR-143 resulted in the prevention of adipocyte differentiation [32]. In addition, let-7a and let-7f (members of the let-7 family) are well-known regulators in development, and in cellular basal metabolism, and are present in abundance in various species including mammals, flies, worms, and plants [33]. Furthermore, the expression levels of the 18 most abundant miRNAs show good correlation (Person's *r* = 0.875±0.121, *n* = 18) between the q-PCR and small RNA-seq sequencing results, which highlights the high confidence of the results obtained using the deep-sequencing approach (Figure S2).

### Extraordinary miRNA expression implied a role for GOM in metabolic risk

The 409 unique miRNAs were divided into six categories according to their tissue-related expression (Table 1 and Table S4). Category 1 consisted of 284 (69.44%) unique miRNAs that were co-expressed in all six ATs. Category 6 consisted of 53 (12.96%) unique miRNAs which were expressed specifically in only one of the six ATs. The total number of miRNAs expressed in more than one and less than six ATs (category 2, 3, 4 and 5) was 72 (17.60%). Notably, the vast majority (29/53, 54.71%) of the tissue-specific miRNAs were specifically expressed in GOM, and included three members of the miR-17-92 cluster (miR-18a-3p, miR-20-3p and miR-19b-1-5p) and two members of the miR-181 family (miR-181a-2-3p and miR-181b-2-3p), both of which are known to be important for the development and production of the pro-inflammatory B-cells and T-cells [34], [35], [36].

**Table 1:** miRNAs classified into six categories according to the status of tissue-related expression.

Category	Number of miRNAs	Defination
1	284	miRNAs co-expressed in six libraries
2	23	miRNAs absent in one library
3	13	miRNAs absent in two libraries
4	17	miRNAs absent in three libraries
5	19	miRNAs absent in four libraries
6	53	miRNAs specifically expressed in only one library

**Figure 2:**
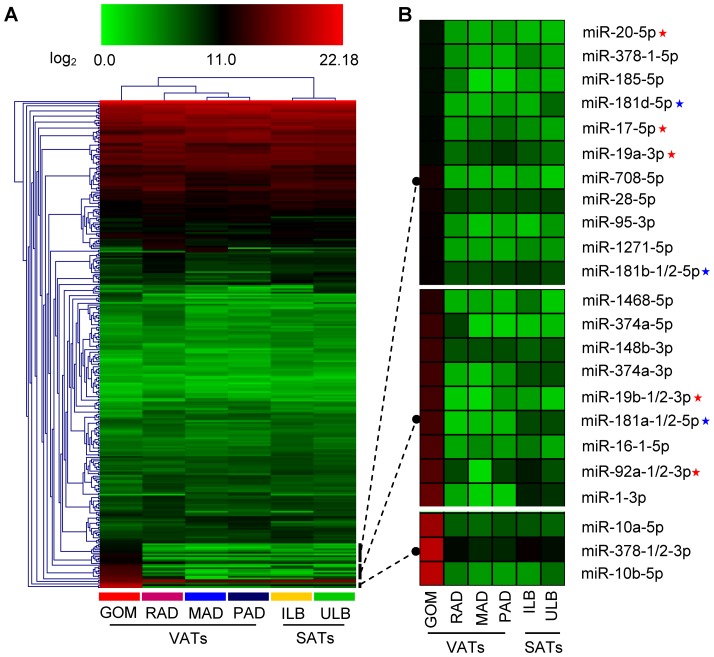
The expression profiles corresponding to 284 co-expressed miRNAs. (A) Hierarchical clustering analysis for six adipose tissues. (B) Three miRNA subgroups that specially enriched in GOM. Red stars : the members of the miR-17-92 cluster. Blue stars: the members of the miR-181 family.

Subsequently, we performed hierarchical clustering based on the expression profiles of the 284 miRNAs that were co-expressed in all six ATs, and observed a deep split between the VATs and SATs. The two SATs (ILB and ULB) were tightly clustered into a subgroup, which were clearly distinct from the four VATs (GOM, MAD, RAD and PAD). This significant variation in miRNA expression may be responsible, at least in part, for the phenotypic differences between the SATs and VATs; for example, their anatomical, functional, and metabolic differences (Figure 2A). Of the four VATs, the GOM was clearly separated from the others with several highly expressed miRNAs (Figure 2B). Remarkably, seven members of the miR-17-92 cluster (miR-17-5p, miR-19a-3p, miR-19b-1/2-3p, miR-20-5p and miR-92a-1/2-3p) and five members of the miR-181 family (miR-181a-1/2-5p, miR-181b-1/2-5p and miR-181d-5p) were highly enriched in the GOM; this cluster and family are both important for the development and production of B- and T-cells [34], [35], [36]. These results are consistent with the fact that milky spots, mainly comprising macrophages and B- and T-lymphocytes, are widely distributed in the GOM [37], [38]. Other GOM-enriched miRNAs were also related to various pathological responses. For example, miR-10a-5p as a vital modulator, could suppress the growth of chronic myeloid leukemia CD34+ cells [39], and miR-10b-5p targets the potent inflammatory mediator *KLF4* (Kruppel-like factor 4) [40] and has important roles in tumor invasion and the initiation of metastasis in breast cancer [41]. These results are consistent with the critical role of abdominal GOM in immune and inflammatory responses [19], [42], [43].

### Inflammation-related miRNAs were specifically enriched in VATs

**Figure 3:**
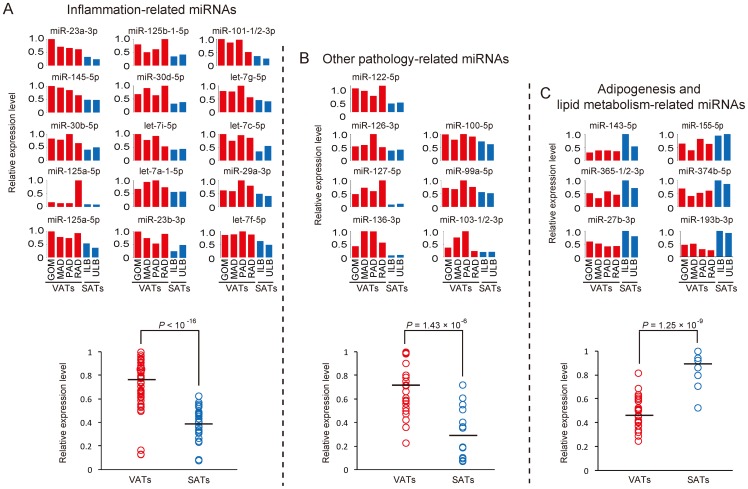
The expression changes of specifically enriched miRNAs across six adipose tissues. (A) The expression pattern of inflammation-related miRNAs across six ATs. (B) The expression pattern of pathology-related miRNAs (other than inflammation-related miRNAs) across six ATs. (C) The expression pattern of adipogenesis and lipid metabolism-related miRNA across six ATs. The red and blue bars/circles represent the relative expression levels of miRNAs for VATs and SATs, respectively. The black lines across the circles present the average expression level for a certain type of miRNAs, and Student's *t* test was used for the testing significance of difference between VATs and SATs.

We performed a pairwise comparison analysis between each of the VATs and SATs for the 284 co-expressed miRNAs, and identified 22 and 21 differentially expressed miRNAs that were specifically enriched in the VATs and SATs, respectively (Table 2 and Table S5). Notably, vast majority of the VATs-specific enriched miRNAs (15/22, 68.18%) were associated with inflammation based on the annotation from the Pathway Central database (SABiosciences, MD, USA) (Figure 3A). MiR-let-7a-1-5p, miR-let-7c-5p, miR-let-7f-5p, miR-let-7g-5p and miR-let-7i-5p (members of the miR-let-7 family) are involved in allergic airway inflammation by suppressing the expression of interleukin-13 [44], and also inhibit cell transformation by directly targeting interleukin-6 [45]. MiR-145, a regulator of inflammation, was reported to be down-regulated in longstanding ulcerative colitis [46]. MiR-30b-5p and miR-30d-5p, two members of the miR-30-family, can inhibit mitochondrial fission by suppressing the expression of tumor protein 53 (*p53*), and can also promote cellular invasion and immunosuppression by targeting GalNAc transferase 7 [47]. MiR-125a-5p is a critical inhibitor of the pro-inflammatory response by targeting oxysterol binding protein-like 9, which resulted in decreased secretion of inflammatory cytokines [48]. MiR-23b-5p suppresses nuclear factor κB (*NF-κB*) activation and inflammatory cytokine expression by targeting *TAB2* (TGF-β-activated kinase 1/MAP3K7 binding protein, *TAB3* (TGF-β-activated kinase 1/MAP3K7 binding protein 3), and *IKK-α* (inhibitor of NF-κB kinase subunit α) [49]. MiR-29a is an important threshold modulator of thymic epithelial cell response to peripheral PAMP (pathogen-associated molecular patterns) signals by targeting *INFAR1* (Interferon-α receptor 1) [50]. By targeting *MKP-1* (MAPK phosphatase-1) miR-101-1-5p is involved in the regulation of the innate immune responses of macrophages to lipopolysaccharide (LPS) [51].

**Table 2:** Identification of VATs- and SATs-specific enriched miRNAs.

Comparison	Up-regulated	Down-regulated
GOM vs. ILB	76	120
MAD vs. ILB	46	155
PAD vs. ILB	52	134
RAD vs. ILB	124	56
GOM vs. ULB	78	109
MAD vs. ULB	57	138
PAD vs. ULB	60	114
RAD vs. ULB	125	41
Specifically enriched miRNAs*	22 (VATs-specific)	21 (SATs-specific)

* :if a differentially expressed miRNA was identified from all eight comparisons with the same regulation pattern, it was termed as a specifically enriched miRNA for VATs or SATs.

In addition to these well-annotated miRNAs, seven other VATs-specific enriched miRNAs (miR-122-5p, miR-126-3p, miR-127-5p, miR-136-3p, miR-100-5p, miR-99a-5p and miR-103-1-3p) were found to be related to various pathological responses (Figure 3B). By modulating cyclin G1, miR-122-5p influences *p53* stability and transcriptional activity and reduces the invasion capability of hepatocellular carcinoma-derived cell lines [52]. MiR-126-3p can inhibit the growth of lung cancer cell lines in vitro and in vivo by down-regulating *VEGF* (vascular endothelial growth factor) [53]. MiR-127-5p and miR-136-3p (members of the miR-433-127 cluster) are involved in hepatocarcinogenesis [54]. MiR-100-5p targets *Plk1* (polo-like kinase 1), a critical regulator of many stages of mitosis, resulting in the inhibition of cancer progression in nasopharyngeal cancer cell lines [55]. MiR-99a-5p, as a potential tumor suppressor, was down-regulated in advanced prostate cancer cell lines relative to the parental cell lines and its overexpression was reported to inhibit the growth of prostate cancer cells and decrease the expression of prostate-specific antigen [56]. MiR-103 decreased early in Alzheimer's disease and accelerated disease progression through the regulation of *BACE1* (β-site amyloid precursor protein-cleaving enzyme 1) [57].

### Adipogenesis and lipid metabolism-related miRNAs were enriched in SATs

In contrast, the SATs-specific enriched miRNAs (Figure 3C) were related mainly to adipogenesis and lipid metabolism. For example, miR-155-5p has been shown to inhibit adipogenesis by targeting the transcriptional factor *C/EBP-β* [58]. MiR-143-5p was elevated in adipogenesis and its inhibition with antisense oligonucleotides prevented adipocyte differentiation [4]. MiR-193b-3p and miR-365 (members of the miR-193-365 cluster) were revealed as central regulators of brown fat differentiation and adipogenesis [59]. MiR-27b-3p is a negative regulator of adipocyte differentiation by suppressing *PPARγ* (peroxisome proliferator-activated receptor γ) expression [8]. In pigs, miR-374b-5p was reported to be involved in the effect of maternal dietary protein on lipid metabolism in the offspring by targeting *C/EBP-β* [60].

### Functional enrichment analysis of the mRNA targets of the differentially expressed miRNAs

**Figure 4:**
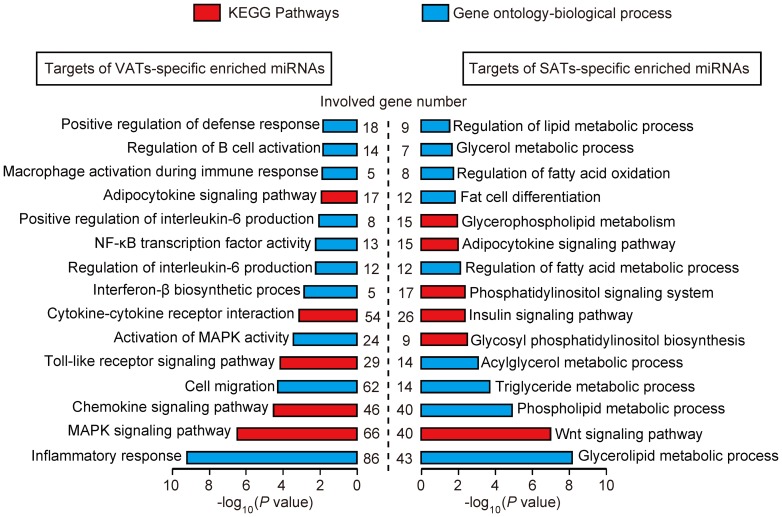
KEGG pathways and Gene Ontology-Biological Processes (GO-BP) enriched for target genes of VATs- and SATs-specific enriched miRNAs. The *P* values was calculated using Benjamini-corrected modified Fisher's exact test.

To further understand the distinct functional features between the VATs and SATs, the target protein coding genes of the miRNAs, which were specifically enriched in the VATs (2,572 mRNA genes) and SATs (1,961 mRNA genes), were predicted using PicTar [26], TargetScan human 6.2 [27], and MicroCosm Targets (version 5.0) [28] (Table S6). The predicted target genes were analyzed using the DAVID software [29] to determine whether they were enriched for specific functional categories and pathways. As expected, the target genes of the VATs-specific enriched miRNAs were associated primarily with immune and inflammatory processes, such as ‘inflammatory response’ (86 genes, *P* = 6.86×10^−10^), ‘chemokine signaling pathway’ (46 genes, *P* = .25×10^−5^), ‘Toll-like receptor signaling pathway’ (29 genes, *P* = 7.84×10^−5^), ‘regulation of interleukin-6 production’ (12 genes, *P* = 6.35×10^−3^), ‘*NF-kB* transcription factor activity’ (13 genes, *P* = 6.51×10^−3^), and ‘macrophage activation during immune response’ (5 genes, *P* = 1.48×10^−2^). In contrast, the target genes of the SATs-specific enriched miRNAs were associated mainly with lipid and energy metabolism, such as ‘glycerolipid metabolic process’ (43 genes, *P* = 7.66×10^−9^), ‘lipid biosynthetic process’ (38 genes, *P* = 7.36×10^−^6), ‘Wnt signaling pathway’ (40 genes, *P* = 1.14×10^−7^), ‘phospholipid metabolic process’ (40 genes, *P* = 1.45×10^−5^), ‘triglyceride metabolic process’ (14 genes, *P* = 2.32×10^−4^), ‘insulin signaling pathway’ (26 genes, *P* = 4.76×10^−3^), and ‘regulation of fatty acid metabolic process’ (12 genes, *P* = 8.94×10^−3^) (Figure 4). These results further suggested that SATs are involved mainly in lipid and energy metabolic homeostasis, whereas the VATs are susceptible to inflammation and should be regarded as a potential metabolic risk factor of obesity.

## Discussion

It have been well documented that the type of adipocytes, their endocrine function, lipolytic activity, response to insulin and other hormones are different between SATs and VATs. The content of non-adipocyte cells from ATs, such as monocytes/macrophages, were also shown to be significant different between SATs and VATs [61], [62]. It was shown that individual tissues maintain a unique miRNA profile, suggesting that miRNAs contribute to specific tissue function by regulating different gene targets [63]. As expected, we found that distinct ATs from different anatomical locations expressed a core number (284/409, 69.44%) of miRNAs that may fundamental for the regulation of genes involved in adipose metabolism (Table 1), but significant differences in miRNA expression were demonstrated. This result suggests that VATs and SATs are two locations of a developmentally homogeneous adipose organ [64].

Currently, it is thought that the increase in VATs rather than SATs best correlates with measures of insulin resistance [65] and cardiovascular diseases [66], and contributed to the chronic low-grade inflammation accompanied by these metabolic disorders in ATs [67], [68], [69]. Growing evidence indicates that deregulation of miRNAs is closely associated with obesity-related metabolic disorders including type 2 diabetes and cardiovascular diseases [70], [71]. Specific miRNAs have been implicated in adipogenesis and mature adipocyte function [32]. Nonetheless, the involvement of miRNAs in adipose tissue inflammation has been scarcely investigated. As yet, only a few miRNAs have been identified as relevant in this field [72], [73]. In this study, we presented a set of inflammation- and pathology-related miRNAs, which were specifically enriched in porcine VATs and could contribute to the special role of VATs in metabolic risk. Further studies are encouraged to validate the role of these miRNAs in metabolic disorders and obesity-associated adipose tissue inflammation in human individuals under physiological and pathological conditions.

In summary, we have presented a comprehensive comparison of miRNA expression among six ATs and focused on the variations in miRNA expression between the VATs and SATs, which reflected the intrinsic differences in their physiological and metabolic roles. Compared with the SATs that were related mainly to adipogenesis and lipid metabolism, the VATs (in particularly, the GOM) were mainly associated with the immune and inflammation responses, and should be deemed as a potential metabolic risk factor of obesity. Our results will benefit future studies into the metabolic role of the distinct AT compartments in obesity-related metabolic dysfunction, and in the further development of the pig model for human metabolic research.

## Supporting Information

Figure S1 Characterization of the small RNA-seq data. (A) Filter process of sequencing data. (B) Size distribution of high-quality reads for six small RNA libraries.
(TIF)

Figure S2 Q-PCR validation for 18 miRNAs with the highest expression level across six adipose tissues. The *Y*-axis on the left represents the percentage of a certain miRNA accounted for in total high-quality reads resulting from small RNA-seq. The *Y*-axis on the right represents the relative expression levels of a certain miRNA derived from q-PCR. Pearson correlation was used to determine the relation of miRNAs expression changes between the q-PCR and the small RNA-sequencing approaches. Values are means ± SD.
(TIF)

Table S1 Primer sequences used in q-PCR validation.
(XLS)

Table S2 Porcine Known and conserved miRNAs identified in this study.
(XLS)

Table S3 Porcine unique miRNAs identified in this study.
(XLS)

Table S4 MiRNA categories defined by tissue-related expression pattern.
(XLS)

Table S5 Expression patterns of VATs- and SATs-specific enriched miRNAs across distinct adipose tissues.
(XLS)

Table S6 Predicted mRNA targets for the VATs- and SATs-specific enriched miRNAs.
(XLS)
