# Supplementary figures and images for: Intrinsic Features in MicroRNA Transcriptomes Link Porcine Visceral Rather than Subcutaneous Adipose Tissues to Metabolic Risk
Source: PLoS One. 2013 Nov 6;8(11):e80041. doi: 10.1371/journal.pone.0080041 (PMC3819305; doi:10.1371/journal.pone.0080041)

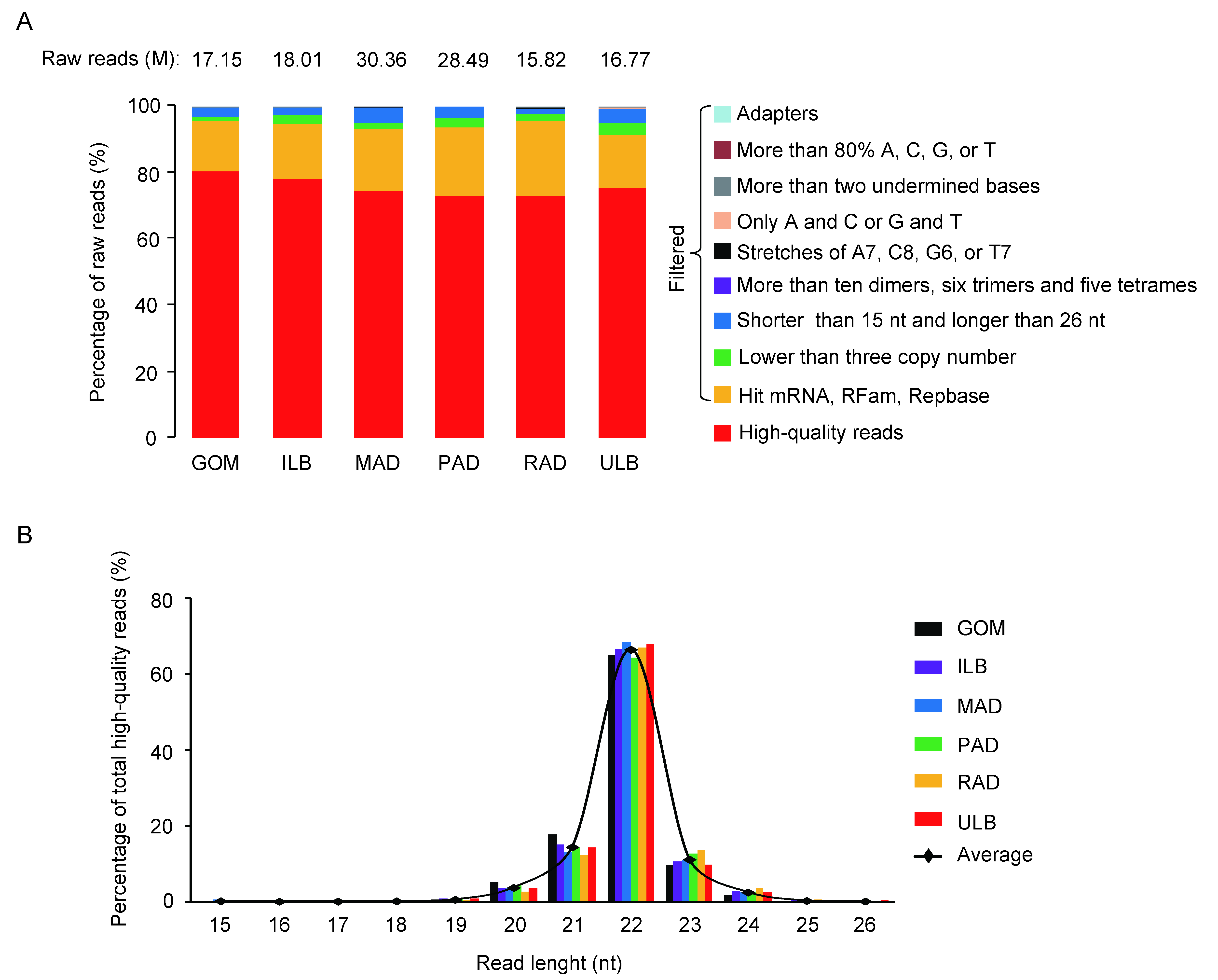

Supplement: Figure S1 — Characterization of the small RNA-seq data. (A) Filter process of sequencing data. (B) Size distribution of high-quality reads for six small RNA libraries. (TIF) [file pone.0080041.s001.tif]

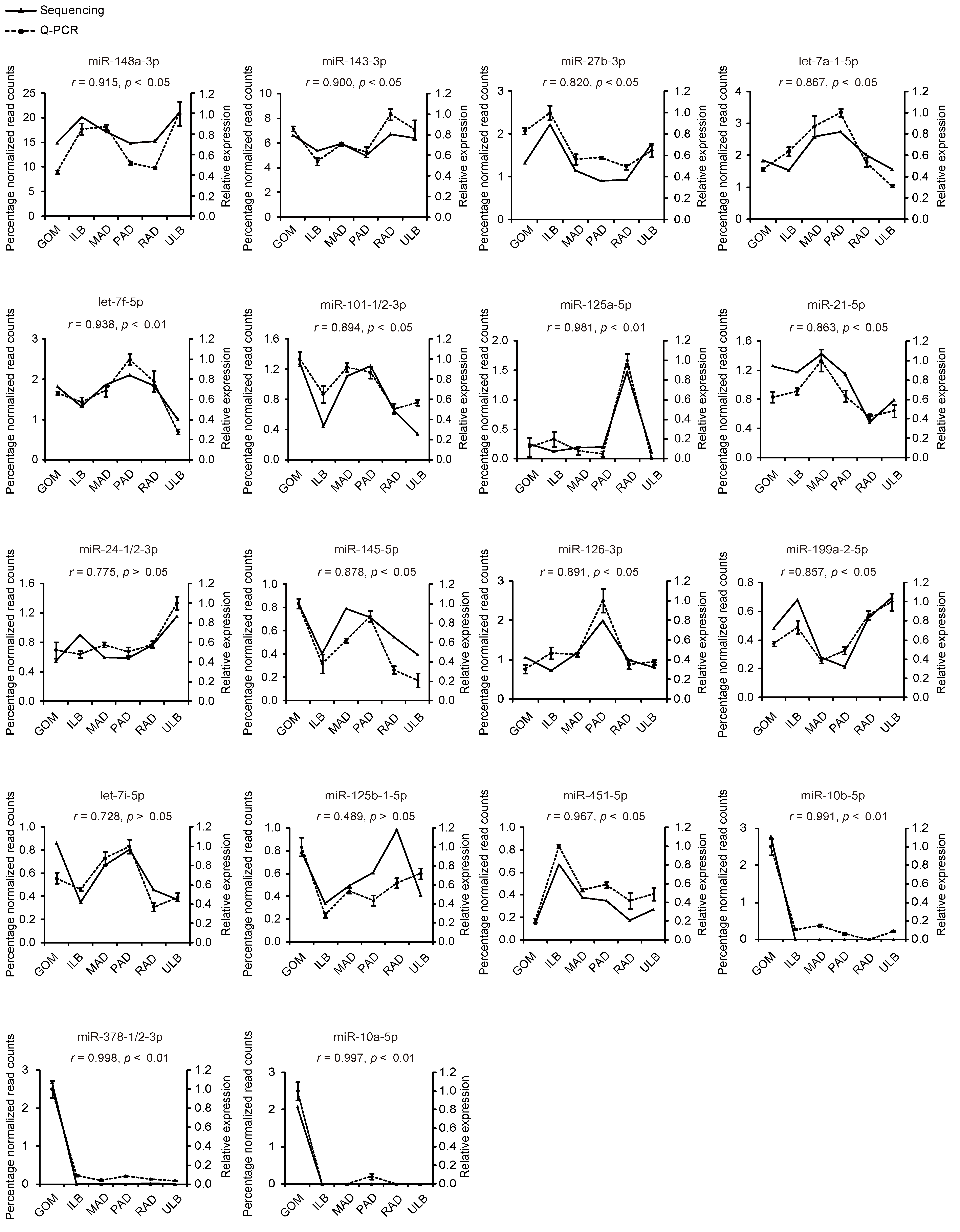

Supplement: Figure S2 — Q-PCR validation for 18 miRNAs with the highest expression level across six adipose tissues. The Y-axis on the left represents the percentage of a certain miRNA accounted for in total high-quality reads resulting from small RNA-seq. The Y-axis on the right represents the relative expression levels of a certain miRNA derived from q-PCR. Pearson correlation was used to determine the relation of miRNAs expression changes between the q-PCR and the small RNA-sequencing approaches. Values are means ± SD. (TIF) [file pone.0080041.s002.tif]
